# Efficiency of Topical Beta-Blockers for Epistaxis Control in Ulcerated Infantile Hemangioma

**DOI:** 10.7759/cureus.67709

**Published:** 2024-08-25

**Authors:** Ngan Lam, Thomas Schrepfer

**Affiliations:** 1 Otolaryngology - Head and Neck Surgery, University of Florida Health, Gainesville, USA; 2 Otolaryngology, University of Florida, Gainesville, USA

**Keywords:** epistaxis, infantile hemangioma, beta-blocker, timolol, infant

## Abstract

Infantile hemangiomas (IHs) are common childhood benign tumors that are generally self-limiting in nature. However, they can lead to complications, including pain, ulceration, or permanent disfigurement. Successful treatment methods include oral and topical beta-blockers.

We report a case of a two-month-old girl, born full-term, who presented with daily epistaxis that began approximately one week after birth. The epistaxis was previously treated with nasal saline drops. Endoscopy revealed an ulcerating hemangioma over the right anterior nasal septum with involvement of the maxillary crest. The remainder of her physical examination was unremarkable, with no concerns for further airway pathologies.

We initiated a trial of topical Timolol 0.5% TID for one month while continuing nasal hygiene with saline spray and Vaseline. Epistaxis ceased after only a few days of Timolol use, and substantial improvement in appearance, bleeding, and nasal crusting of the right anterior septum was noted. However, the hemangioma was found to be extending into the anterior soft tissue, including the oral vestibulum. Imaging was obtained, and systemic treatment with propranolol was added to her regimen.

The mainstay and first-line treatment for IHs currently consists of topical beta-blockers (such as Timolol) and oral propranolol. In this case, the topical application of Timolol effectively controlled the bleeding and restore function but had limited impact on overall tumor regression, necessitating additional systemic therapy. Although systemic treatment may not always be readily available or may be limited due to potential side effects, early use of topical beta-blockers can be considered a relatively simple and safe first-line treatment option for controlling the symptoms of an intranasal IH in the early life of an infant.

## Introduction

Infantile hemangiomas (IHs) are one of the most common benign tumors of childhood, occurring in approximately 4%-5% of all infants [1,2]. These vascular tumors predominantly affect white neonates, girls, twins, and infants born prematurely or with low birth weight [1]. Although IHs are generally self-limiting, they can lead to complications such as pain, ulceration, or permanent disfigurement if left untreated [3]. The current clinical practice guidelines for managing IHs include oral propranolol, topical Timolol, surgical intervention, and/or laser treatment [1,4]. Recent studies have continued to explore the effectiveness of topical beta-blockers, particularly in challenging anatomical locations, with promising results [5,6].

This case report focuses on the efficacy of topical Timolol for controlling epistaxis in a two-month-old infant with an ulcerated IH involving the nasal vestibulum and septum. The use of topical beta-blockers, like Timolol, has become a favored treatment option due to its localized effect and reduced systemic side effects compared to oral beta-blockers [2,5]. However, the extent of Timolol's effectiveness in controlling bleeding and promoting regression in ulcerated IHs remains under-explored, particularly in challenging anatomical areas such as the nasal cavity. This report aims to contribute to the understanding of topical beta-blockers in treating IHs and highlight the potential need for systemic therapy in cases where topical treatment alone may be insufficient.

## Case presentation

An eight-week-old full-term, otherwise healthy infant presented with recurrent daily epistaxis from the right nostril, which began approximately one week after birth. The epistaxis had previously been managed with nasal saline drops. There were no concerns for impaired nasal breathing, stridor, or possible compromised airway, nor was there a family history of IHs. No imaging had been performed before the initial presentation.

**Figure 1:**
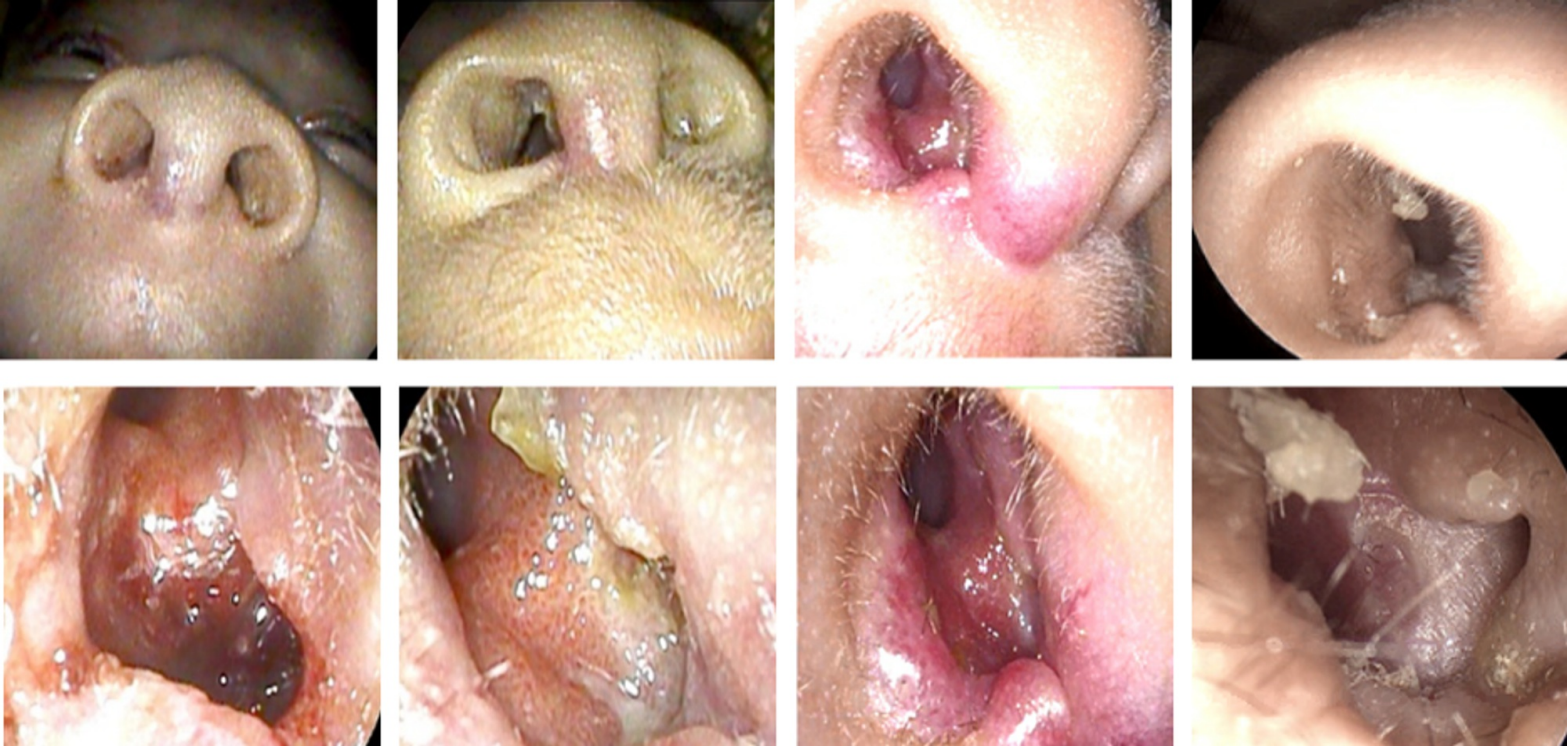
Nasal endoscopy finding of ulcerating hemangioma over right anterior septum on initial presentation, two weeks post-topical Timolol treatment, one-month post-topical Timolol treatment, and final presentation at eight months.

At the initial presentation, examination using flexible nasal endoscopy revealed an ulcerating hemangioma over the right anterior nasal septum with involvement of the maxillary crest (Figure 1). It was recommended that the patient continue nasal hygiene management with saline spray and Vaseline and begin a trial of topical Timolol 0.5% TID for one month.

First post-intervention (two weeks)

After two weeks of using the Timolol drops, epistaxis had ceased. Substantial improvement in the appearance, bleeding, and nasal crusting of the right anterior septum was noted. According to feedback provided by the parents, the bleeding stopped after just a few days of treatment.

Second post-intervention (four weeks)

**Figure 2:**
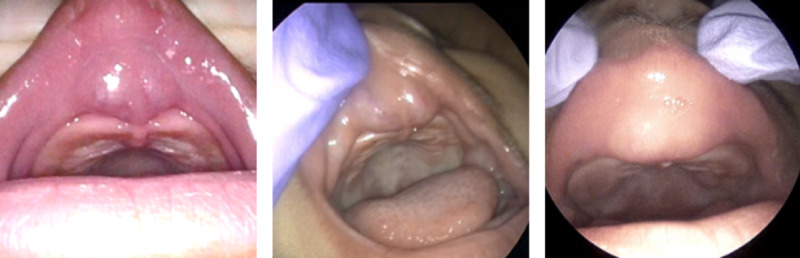
Infantile hemangioma found to involve upper oral vestibulum. Findings and progression at one, three, and eight months.

At four weeks, the hemangioma showed further improvement in appearance. However, we identified that the hemangioma was extending into the oral vestibulum/upper lip and upper jaw (Figure 2). Due to the limited to no effect on tumor regression, the patient was referred to pediatric cardiology for the management of systemic beta-blocker propranolol (Inderal). Additionally, magnetic resonance imaging (MRI) was ordered to assess the extension of the oral vestibulum mass and to rule out intracranial abnormalities.

Follow-up (eight months)

**Figure 3:**
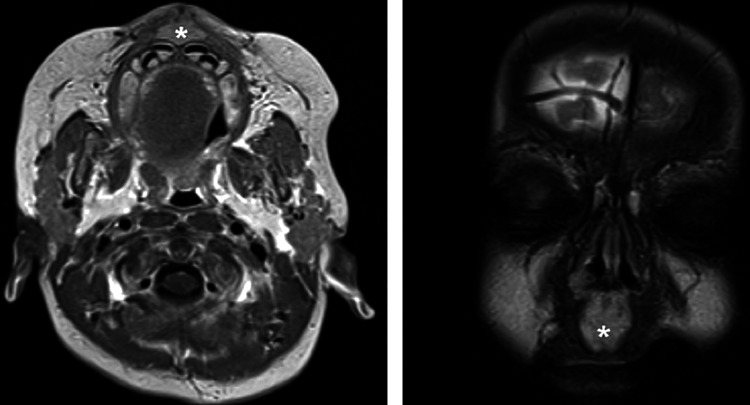
MRI of the head (left) with and (right) without contrast. Findings are consistent with proliferative hemangioma of the premaxilla (*), at the level of the nasal labial folds without the involvement of the oral cavity.

After a total of eight months, the patient returned for follow-up. During this period, an MRI was performed, revealing the extent of the oral vestibulum mass and ruling out intracranial abnormalities (Figure 3). The patient also underwent systemic beta-blocker therapy with propranolol due to the hemangioma’s persistence. Unfortunately, we were unable to continue monitoring the patient after this follow-up due to the family’s relocation.

## Discussion

IHs are the most common benign tumors in infancy, often requiring intervention when complications, such as ulceration, pain, or potential disfigurement, arise. In this case, we described an infant with an ulcerated IH located on the nasal septum, a particularly challenging site due to the risk of bleeding, ulceration, and potential distortion of anatomical landmarks.

The topical application of beta-blockers, specifically Timolol, has gained favor as a first-line treatment for superficial and ulcerated IHs due to its localized effects and minimal systemic side effects. Timolol, a non-selective beta-blocker initially used in the treatment of glaucoma, has shown efficacy in reducing the size and symptoms of IHs through mechanisms that include vasoconstriction, decreased expression of angiogenic factors, and induction of apoptosis in capillary endothelial cells [6,7]. In our case, the use of topical Timolol effectively controlled the epistaxis within a few days and significantly improved the appearance of the ulcerated hemangioma over the initial four-week period. However, despite these improvements, the treatment had limited effects on the overall regression of the tumor, necessitating the addition of systemic therapy with propranolol.

Propranolol, an oral beta-blocker, is the most established systemic treatment for IHs and has revolutionized the management of these lesions since its introduction [8]. However, concerns about systemic side effects, particularly in infants, often lead to the initial use of topical treatments like Timolol. In our patient, the decision to transition to systemic therapy was made after the hemangioma showed persistent growth into the oral vestibulum despite the use of Timolol. This highlights the potential limitation of topical therapy in cases where the hemangioma is extensive or rapidly proliferative, as supported by recent studies that suggest combining both topical and systemic therapies for more complex IH cases [7,9].

While our case supports the efficacy of topical Timolol for controlling symptoms such as bleeding in ulcerated IHs, it also underscores the importance of close monitoring and the potential need for systemic therapy in cases with limited response to topical treatment. The patient was initially observed at two and four weeks, with significant symptomatic improvement noted, but persistent tumor growth required further intervention. This aligns with recommendations from recent studies suggesting that more frequent monitoring during the first month of treatment, such as at weekly intervals, could provide a better understanding of the treatment’s effectiveness and inform timely adjustments [6,10].

The limitations of this case include the lack of more frequent follow-up during the initial treatment period and the inability to continue monitoring the patient after eight months due to the family’s relocation. These limitations highlight the need for future studies to document the treatment progress at more regular intervals and to explore the long-term outcomes of combining topical and systemic beta-blockers in treating complex IHs.

## Conclusions

In conclusion, while topical Timolol can be a safe and effective first-line treatment for controlling symptoms of ulcerated IHs, especially in settings where systemic therapy may be limited, its use should be carefully monitored, and systemic therapy should be considered if there is no significant tumor regression. Early referral and multidisciplinary management, including collaboration with pediatric cardiology for systemic beta-blocker therapy, are crucial in optimizing outcomes for infants with complicated IHs.
